# Disclosing Inflammatory Bowel Disease: A Systematic Review and Meta-Synthesis Exploring the Experience of, and Barriers and Facilitators to, Self-Disclosure

**DOI:** 10.1007/s10880-025-10079-z

**Published:** 2025-06-02

**Authors:** Emma Harriman, Fergal W. Jones, Alexa Duff

**Affiliations:** 1 Canterbury Christ Church University, Canterbury, UK; 2 Surrey and Borders Partnership NHS Foundation Trust, Leatherhead, UK; 3 London North West University Healthcare NHS Trust, London, UK

**Keywords:** Inflammatory bowel disease (IBD), Crohn’s disease, Ulcerative colitis, Self-disclosure, Stigma

## Abstract

Due to its invisible nature, individuals must make decisions around self-disclosing Inflammatory Bowel Disease (IBD). Existing reviews have explored stigma as a barrier to disclosing IBD; however, other factors potentially affecting disclosure decisions have not been recently reviewed. The current review sought to synthesise qualitative papers exploring the experiences of disclosure, and identify facilitators and barriers to disclosure, within IBD. A systematic search identified 34 journal articles, comprising 1,004 participants. Following critical appraisal, a thematic meta-synthesis was completed. (PROSPERO registration ID CRD42023481441). A total of six main themes relating to self-disclosure were identified: it’s a stigmatised illness; a wish to conceal; difficulties discussing IBD; a need to disclose; balancing the need to disclose and the reluctance to disclose; and varied consequences to disclosure. Overall, the included studies were adequate quality; however, weaknesses in reflexivity were observed across a high proportion. This review highlights the complexity of disclosure decisions and describes how personal beliefs, social contexts, and previous experiences can influence these. Potential clinical implications, the impact for public health services, and the need for future research are discussed.

**Supplementary Information:**

The online version contains supplementary material available at 10.1007/s10880-025-10079-z.

## Introduction

Inflammatory Bowel Disease (IBD) is a progressive autoimmune disease encompassing Crohn’s disease (CD) and Ulcerative Colitis (UC). This lifelong condition (affecting approximately 0.81% of the UK population) presents as a relapsing–remitting disease (Liverani et al., 2016) causing periods of unpleasant symptoms, including abdominal pain, difficulties with bowel movements (frequent diarrhoea, constipation, or blood), vomiting, fatigue, weight loss, and growth difficulties (Farrell et al., 2016), which can be challenging for individuals living with the disease. Like other chronic illnesses, IBD is often considered an “invisible illness,” as symptoms are less outwardly detectable by others (Donoghue & Siegel, 2000; Joachim & Acorn, 2000; Stone, 2005). Due to its invisibility, individuals living with IBD often outwardly appear “healthy” (Micallef-Konewko, 2013; Vickers, 1997), resulting in them having to make decisions about self-disclosing their illness to others.

Although sharing general information about oneself with others has been demonstrated to positively impact physical health, mental health, and social relationships within general social settings (Omarzu, 2000), the choice of what information, when, and how much, to share with others is a personal one. When people determine whether to disclose a concealable illness, the Disclosure Processes Model (DPM, Chaudoir & Fisher, 2010) proposes a complex interaction of personal and social contextual factors, which determines *when* and *why* to disclose, including the type and severity of illness, and access to social support (Benson et al., 2015; Chaudoir et al., 2011; Greene, 2000; Vickers, 1997).

### Disclosing a Stigmatised Illness

According to the DPM, the experience of stigma, or the perceived risk of stigmatisation, may prevent the self-disclosures of invisible illnesses, including HIV, epilepsy, and cancer (Catona et al., 2016; Clifford et al., 2023; Gray et al., 2000; MacLeod & Austin, 2003; Wanjala et al., 2023). For IBD specifically, fears of stigmatisation due to the physical symptoms (Daniel, 2002; Taft et al., 2009) have been associated with psychological distress, reduced health-related quality of life, reduced adherence with medication, and decreased self-esteem (Taft & Keefer, 2016). It has been reported that individuals with IBD may attempt to conceal their illness in attempt to “pass” as someone without a chronic illness, and therefore, avoid the perceived stigmatisation (Taft & Keefer, 2016).

In a recent qualitative review, Muse et al. (2021) identified stigma as a common experience for people with IBD, with individuals feeling “labelled” by the disease and experiencing a “loss of self” following the diagnosis. In this review, the authors refer to the impact the stigmatisation of IBD has on disclosure decisions, with participants wanting to be understood, but feeling the need to conceal their illness identity. Despite the potential benefits from disclosing their IBD, it was found that the fear of stigmatisation and shame prevented individuals feeling safe to talk about their illness. Guo et al. (2020) also reviewed the experience of stigma on self-disclosing IBD, highlighting the different experiences individuals’ have when talking about their diagnosis across different social contexts, including young people feeling forced to “explain” their behaviours and make workplace disclosures to access appropriate “sick leave”.

Although these existing reviews provide an understanding of how stigma impacts self-disclosure for individuals with IBD, their focus on stigma does not consider other factors which may be important in contributing to these decisions. An earlier review by Micallef-Konewko (2013), that sought to explore IBD disclosure in young people more broadly, identified the risk of bullying, uncertainty around the diagnosis, and a desire to live a normal life, as barriers to disclosing a diagnosis. However, due to the sparsity of research at that time, the review predominantly drew from other chronic illness literature, including HIV, sickle cell disorder, and cancer diagnoses, and applied it to IBD, rather than identifying the experiences of IBD directly. Therefore, a new review is needed.

### Current Review

Disclosing physical health conditions involves complex interpersonal and intrapersonal processes (Woodgate et al., 2022), which can have both positive and negative effects on the individual. To date, however, the experiences of disclosing IBD across the life span and the barriers/facilitators associated with disclosure for this population have not been adequately reviewed, despite the potential impact this understanding may provide for the psychological care of individuals living with IBD. Therefore, this systematic review and meta-synthesis aimed to understand the experiences, barriers and facilitators, associated with disclosure decisions for individuals living with IBD. This review focussed on qualitative research due to these methods offering more in-depth detail about individual experiences compared to quantitative methodologies.

## Method

The form of meta-synthesis adopted was thematic synthesis due to its appropriateness in analysing experiences, facilitators, and barriers within healthcare literature (Barnett-Page & Thomas, 2009; Thomas & Harden, 2008). This review was registered with The International Prospective Register of Systematic Reviews (PROSPERO, registration ID CRD42023481441).

### Search Strategy

Searches of electronic databases PsychInfo; Medline (Ovid); Scorpus; ASSIA; and CINAHL Complete (EBSCO) were completed in October 2023, using pre-planned search terms (see Supplementary Material). Following the introduction of Infliximab in 1999, the outcomes and experiences of people living with IBD improved (Feagan et al., 2007). Related changes likely included disease activity being better controlled and the side-effects of treatment being less obvious, potentially decreasing the visibility of the illness and hence potentially influencing decisions about, and experiences of, self-disclosure. Therefore, this review focusses on studies published from 2000 onwards, after the introduction of Infliximab.

### Eligibility Criteria

Study eligibility was determined by the application of the criteria in Table 1.

**Table 1:** Inclusion and exclusion criteria for papers

	Criteria
Inclusion	- Participants had a diagnosis of IBD (Crohn’s disease or ulcerative colitis) - Employed a qualitative approach to methodology and data analysis (the qualitative part of mixed methods studies were included if the qualitative results were relevant to the review) - Explored experiences of disclosing/sharing/discussing/talking about an IBD diagnosis or living with the disease (studies were not required to have explored disclosure as the focus of the research but were included if some of their findings were relevant to this review) - Peer reviewed journal articles or published theses/dissertations - Written in English
Exclusion	- Studies reporting the experiences of friends, parents, or family members of people with IBD - Studies containing participants with different physical health conditions (including other gastrointestinal disorders), where the results were not presented independently for those with IBD

### Screening and Selection

**Fig. 1:**
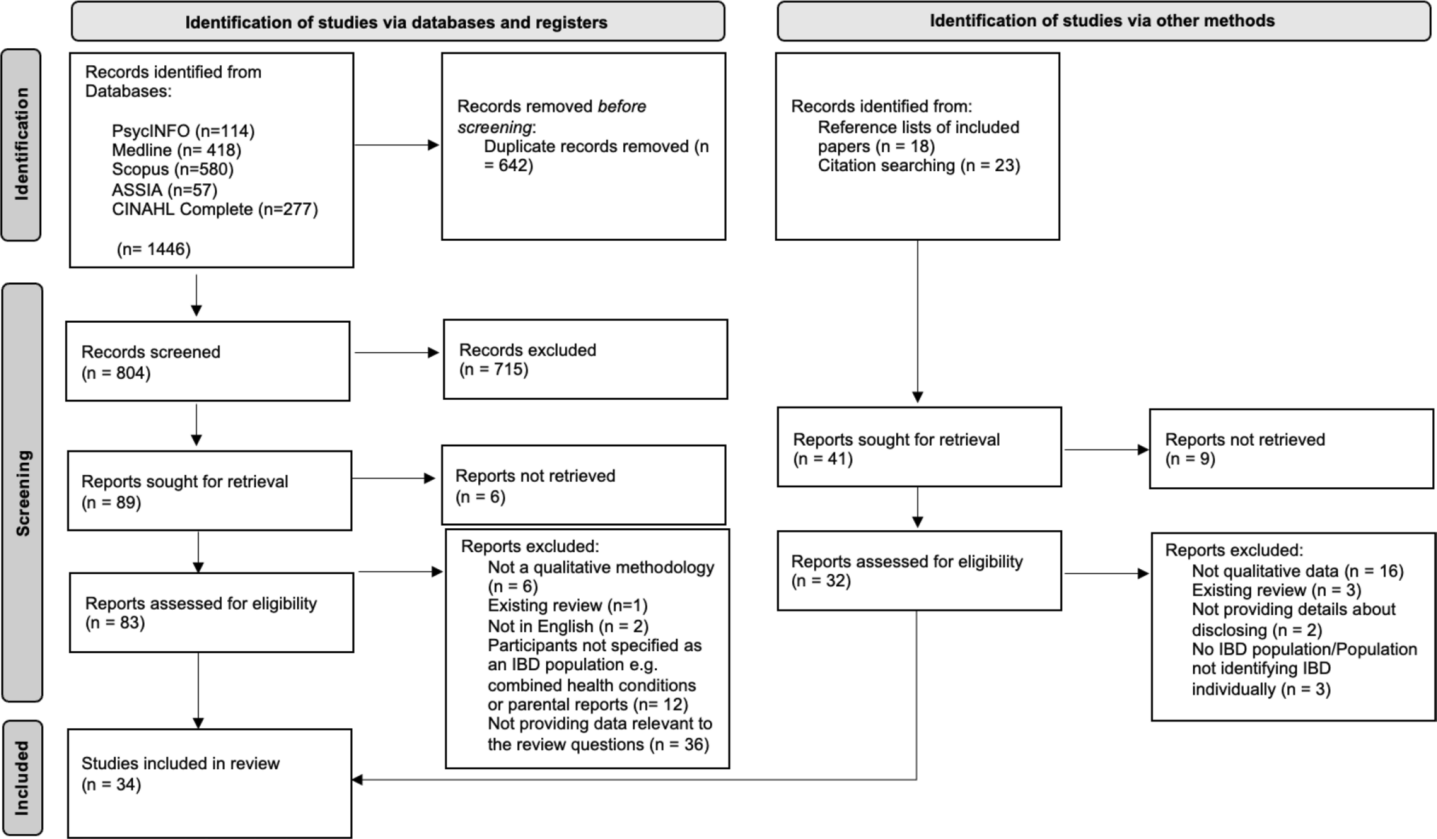
PRISMA flow chart for study selection

This review followed the Preferred Reporting Items for Systematic reviews and Meta-Analyses – PRISMA (Moher et al., 2009) which is summarised in Fig. 1. Screening of records occurred in two stages: (i) if articles clearly did not meet the inclusion criteria based on their title and abstract, they were excluded; and (ii) all remaining articles had their full text reviewed for eligibility. Forward and backward searching of included studies was also conducted. An independent reviewer screened 20% of the included studies against the eligibility criteria, with 85.7% inter-rater reliability.

### Quality Appraisal

Study quality was assessed by the lead author using the Critical Appraisal Skills Programme framework for qualitative studies (CASP, 2018). An independent reviewer appraised a random 50% of the studies. The inter-rater quality agreement was 85%. Most disagreement occurred between ratings of “can’t tell” and “no” and was resolved following discussion.

### Approach to Synthesis

All qualitative results that provided information relevant to the research questions were extracted and inputted into NVIVO 12 for analysis. A thematic analysis was conducted by the lead author following the steps outlined by Thomas and Harden (2008): developing “free codes”, organising codes into descriptive categories, and developing analytic themes. Free-codes were initially developed through line-by-line coding. This bank of codes was organised and grouped based on similarities and differences. The final stage of synthesis involved applying meaning to develop analytical themes, which was completed through discussions by all authors.

### Reflexivity

Braun and Clarke (2019) highlight the subjectivity of thematic analysis, with researchers’ assumptions influencing the analytic process. Throughout the research, the authors considered their prior experiences and expectations, discussing these to minimise their influence on the analysis. Please see the Supplementary Material for further details.

## Results

### Overview of Included Studies

The 34 included studies are summarised in Table 2. Studies varied in their number of participants (ranging from 6 to 134) and included participants from across the lifespan (6- to 78-year-olds). Many studies did not include details on ethnicity (*n* = 24). Although most studies included a sample of participants with different types of IBD, four looked independently at CD (Kitchen et al., 2020; Ruan & Zhou, 2019; Wåhlin et al., 2019; Wang et al., 2023), one looked at UC (Colmer, 2021), and two looked at experiences of individuals either with, or who had previously had, a stoma (Sammut et al., 2017; Savard & Woodgate, 2009). Most studies used interviews as their data collection method, with some supplementing with other qualitative approaches. Studies used a range of analysis, with thematic analysis (*n* = 13) and phenomenological approaches (*n* = 7) being most frequent.

**Table 2:** Overview of included studies

Study number	Author (year)	Country	Aims	Participants	Data collection	Qualitative analysis	Key findings relating to disclosure
	Barned et al. (2016)	Canada	To understand how children and adolescents decide whether to conceal or disclose their illness and how they decide when the appropriate time is to tell others. To understand the main challenges faced when disclosing their illness to others	25 participants (13 boys, 12 Girls) aged between 10 and 17	Semi-structured interviews	Thematic analysis	Disclosure decisions were a key part of a young person’s experience of IBD. Several factors including severity of illness, knowledge of IBD, and others asking influenced these decisions
	Carter et al. (2020)	UK	To explore experiences of disclosing an IBD disclosure, in the context of friendships and social connectedness among young people with IBD	31 participants (16 male, 15 female) aged between 14 and 25 (mean age18.7 years). Age at diagnosis ranged from 8 to 23	Conversational interviews, friendship maps and photographs (photo elicitation technique)	Interpretive Description	Decisions about telling friends about having IBD are challenging for many young people. Having control over disclosure is not always possible, and the potential consequences can feel risky. However, most young people had positive experiences of disclosure and gained support from friends and romantic partners
	Colmer (2021)	Holland	To explore what costs and benefits employees with IBD experience because of their disclosure decision	93 participants (23 male, 70 female) with an average age of 34.18 years	Open-ended survey questions	Cutting and sorting technique	Disclosure was associated with little cost and the psychological benefits of transparency and understanding were stated by the participants
	Devlen et al. (2014)	USA	To understand IBD and its treatment from the patient perspective	27 participants (14 male, 13 female) aged between 20 and 59 (Mean age = 31.5). There were 21 participants with UC and 6 with CD	Focus groups and one-on-one interviews	Grounded theory	Disclosure was a major hurdle to overcome, with it being difficult to tell new friends and potential partners about an IBD diagnosis
	Dibley et al. (2017)	UK	To explore stigma experiences in people with IBD	40 participants aged 23–78 (65% female) and 22 (55%) had CD	Unstructured interviews	Interpretative Phenomenological Analysis	Feeling stigmatised was a common experience for participants. However, emotional control, social support, and mastery over disease can support stigma reduction. Although some individuals attempt to conceal their disease due to the risk of others not understanding, self-disclosure had been successful and enabled individuals to receive support and obtain control over their disease
	Dibley et al. (2020)	UK	To explore the experience and meaning of kinship stigma in people with IBD	18 participants (77% female) aged 21 to 64	Unstructured interviews	Iterative Hermeneutic Phenomenology	The response from some family members made individuals feel that they could not talk about their IBD and that attempting to disclose often made them feel misunderstood or dismissed
	Frohlich (2014)	USA	To explore how people with IBD experience stigma because of their disease	14 participants (7 male, 7 female), aged between 20 and 56 years (mean = 23.6). Average age of disease diagnosis was 22.1 (ranging 5–45 years)	Interviews	Phenomenological approach	Most participants perceived their disease to be stigmatising at one point. However, their experiences of disclosing were generally positive
	Gelech et al. (2021)	Canada	To explore how individuals living with IBD make sense of changes in their approach to coping over time	6 young adults (3 female, 3 male) aged 21–28. All diagnosed with IBD between 3 and 10 years prior to study	Semi-structured Interview	Syntactic and thematic analysis	Being more open to friends about their diagnosis and illness was important in participants ability to cope, as it allowed participants to develop and keep important relationships
	Hall et al. (2005)	UK	To gain an understanding of the perspectives and experiences of individuals with IBD and a poor quality of life	31 participants (19 female and 12 male). There were 17 participants with UC and 14 with CD	Individual interviews and 3 Focus groups (male group, female group and a mixed group)	Grounded theory	IBD diagnosis and symptoms were often kept private due to fears of not being understood, embarrassment, fear of being labelled or a burden. This also maintained a sense of "normality" for those living with IBD. However, it was identified that disclosure to others also living with IBD was a positive experience
	Kitchen et al. (2020)	USA	To understand adult and adolescent patients’ experiences of CD, including CD-related symptoms, the burden of living with CD, as well as the symptoms that drive patients to seek medical treatment	Round 1: 24 participants (12 male, 12 female) aged 14 to 75. Round 2: 6 adults (2 male, 4 female) aged 41–74	Interviews	Thematic analysis	Due to the embarrassment around bathroom use, participants avoided telling people, unless an emergency meant that they had to
	Kluthe et al. (2018)	Canada	To elicit perspectives following a diagnosis of Inflammatory Bowel Disease (IBD)	18 participants (7 female, 11 male) aged between 6 and 17. There were 12 diagnosed with CD, 5 with UC and 1 with IBD unclassified	Interviews	Qualitative content analysis	Children varied widely in who they told about the disease. For some, it was inevitable that they would have to tell people. However, others feared sharing the diagnosis because of the threat of teasing. Children experienced a range of responses when they did disclose, including curiosity, understanding, and teasing
	Lolli (2022)	USA	To explore how patients make sense of and communicate their changed relationships to food following an IBD diagnosis	15 female participants aged between 18 and 40. Time since diagnosis ranged from 1.5 years to 28 years	Compassionate interviewing	Thematic analysis	Social situations involving food often led to people feeling pressured into disclosing in attempt to avoid or lessen disapproval from others around their food choices
	Matini and Ogden (2016)	UK	To explore the notion of adaptation in patients with IBD, particularly focussing on lived experiences from diagnosis to the present	22 participants (14 females, 8 male) aged 19–60 years. There were 10 individuals with CD and 12 with UC	Semi-structured Interview	Thematic analysis	Self-disclosure can have a positive impact on relationships for those with IBD because it makes people feel closer and more open with others. Additionally, the misconceptions around IBD as an "invisible" disease make disclosures more necessary
	Micallef-Konewko (2013)	UK	To gain an understanding of what it is like to disclose and talk about IBD as a young person following the transition to secondary school	7 participants (4 males, 3 female) aged 12–13	Semi-structured Interview	Interpretative Phenomenological Analysis	Disclosure was experienced as a risky but potentially rewarding experience, with participants weighing-up potential rewards against anticipated costs. Disclosure was viewed to influence how young people accepted their IBD diagnosis
	Murphy et al. (2022)	United Kingdom	To explore the link between IBD and psychologically difficult emotions and their impact on illness disclosure decisions	16 Females	Interviews	Interpretative Phenomenological Analysis	Women identify that shame is a key emotion linked to their IBD due to it being an "invisible" illness, which can make it difficult to disclose. Women identified finding it more difficult to disclose depending on the person they were telling and identified the difference between voluntary disclosure and times where it felt more of a necessity
	Nehasil (2014)	USA	To discover how individuals participating in a Montana-specific, online support community for those with IBD describe their experiences within the community, and how these experiences have affected their health-related quality of life (HRQOL) in the areas of social support and illness knowledge	10 participants (8 females, 2 male) aged between 20 and 66. There were 7 participants with CD and 3 with UC	|Interviews	Thematic analysis	An online community made it easier for individuals to talk about their illness and feel listened to
	Nicholas et al. (2007)	Canada	To understand the lived experience of/and elements of quality of life as depicted by children and adolescents with IBD	80 young people (44 male, 36 female) aged 7 to 19 years of age (mean age of 13.3 years). The majority had CD (*n* = 61)	Semi-structured Interview	content analysis	Participants reported withdrawing from others to avoid negative judgements and feeling different to them. Fear around other’s reactions and perceptions towards the disease prevent people disclosing about their illness
	O’Leary et al. (2020)	UK	To understand how therapeutic outcomes are realised through the technological features offered by social media platforms	38 participants (20 female, 18 male). There were 25 participants with CD, 13 with UC	Interviews	Deductive thematic analysis	The availability of closed groups contributed to a "safe" space that enabled users to talk openly about their illness and experiences away from their family members. The ability to post-anonymously encourages self-disclosures by reducing the risk of stigma
	Palant and Himmel (2019)	Germany	To determine whether patients with IBD experienced negative effects from social support and if so, how these experiences can be categorised and what role different sources of social support play	42 participants (54% female) aged between 18 and 76 (mean age = 42). Duration of illness ranged from 5 to 40 years	Narrative interviews	Grounded theory	There were several negative effects from social support identified, including unwanted confrontation and undesirable reactions. This included participants experiencing pity from those that they are close with when choosing to disclose their diagnosis
	Peters and Brown (2022)	UK	To examine the relationship between illness identity and self-management of IBD	134 participants (102 females, 31 males, and 1 other gender) aged 19–75	Two open-ended questions	Thematic analysis	Disclosing information about IBD was viewed positively, with it being used as a method of support and to meet other people experiencing similar difficulties
	Restall et al. (2016)	Canada	To illuminate the commonalities of experience, identify variations, and highlight implications for practice, research, and policy, to inform a broader goal of minimizing work disability for people living with IBD	45 participants (23 women, 22 male) aged 21 to 73. Mean disease duration was 10.9 years	Interviews	Thematic analysis	The decision about whether to disclose to an employer or college at work is conflicted, with it being viewed as both potentially helpful and a show of weakness which may result in negative consequences
	Robertson et al. (2022)	UK	To explore the experience of self-conscious emotions in people with IBD and understand the psychological and social impact of self-conscious emotions on individual’s lives	15 participants (4 male, 11 female) aged 25–75. Time since diagnosis ranged from 4 to 20 years	Interviews	Thematic analysis	Talking about IBD as a disease and its accompanying symptoms was viewed as socially unacceptable and something that should be avoided due to other people not being able to tolerate it
	Rouncefield-Swales et al. (2020)	UK	To explore young people with IBD’s friendships and their friendship networks	31 participants (15 female, 16 male) aged between 14 and 25	Interviews, friendship maps and photographs	Interpretive Description	Some young people in the current study concealed their IBD from friends, while others downplayed the seriousness of their condition. Limiting disclosure and explanations about the “gory detail” are aimed at both protecting their friends and minimising the risk of rejection. For young people, some friendships were improved because of disclosure
	Ruan and Zhou (2019)	China	To explore the illness experiences of patients with Crohn’s disease in China and construct an interpretative understanding of these experiences	31 participants (17 males, 14 females) aged 19–68	Interviews	Grounded theory	There were several advantages and disadvantages to disclosing identified by participants. When advantages outweighed the disadvantages or vice versa, the decision to disclose became easier. However, participants still had choices to make regarding the disclosure strategy, including how much to tell and when
	Salazar and Heyman (2014)	USA	To examine the benefits of attending an IBD-specific camp	A total of 25 participants (16 girls and 9 boys) aged between 8 and 17	Interviews, participant observations and fieldnotes	Thematic analysis	Attending the summer camp offered people the opportunity to be around people that were like them and who understood what they were trying to talk about in relation to their disease
	Sammut et al. (2017)	Malta	To explore the experiences of individuals living with an ileoanal pouch	10 participants (6 female, 4 male) aged 25–65. The mean time since formation of the ileoanal pouch was 5.4 years	Semi-structured interviews	Interpretative Phenomenological Analysis	Participants were afraid of how others would react to their experiences, if they found out about them
	Saunders (2014)	UK	To investigate young adults’ representations of IBD-related stigma and explore how this influences their self-disclosure	16 participants (10 female and 6 male) all aged 18–29 years	Interviews	Rhetorical discourse analysis	Participants identify that the stigma associated with IBD, and the taboo nature of symptoms, contributes to concealing their illness from others. However, these accounts identified that some people feel safe talking to those they trust
	Savard and Woodgate (2009)	Canada	To understand the lived experience of young people living with IBD and an ostomy	6 participants (5 women, 1 male), aged between 19 and 24. Time since diagnosis ranged from 3 to 13 years and time since having ostomy ranged from 1 to 8 years. All participants were Caucasian	Interviews	Hermeneutic Phenomenology	The symptoms and bodily changes experienced from treatment and ostomy impacted how much participants felt comfortable disclosing. IBD and treatment was viewed as an illness that is not talked about and an embarrassing thing to discuss
	Schwenk et al. (2014)	USA	To investigate how college-enrolled students with IBD conceptualize and manage their disease and how their experiences of going to college shape their health and health care behaviours	15 participants (7 male, 8 female) aged 19–21. There were 6 with UC and 9 with CD	Interview	Thematic analysis	Participants were guarded about discussing their IBD with other students. Disclosure was often prompted by others being curious about their behaviours. Despite participants being cautious, no negative consequences were reported, rather it allowed developments in social connections
	Vaughan and Jolliffe (2023)	UK	To explore the working lives of those living with the condition IBD	7 participants (4 UC, 3 CD)	Semi-structured interviews	Content analysis	Disclosing within the workplace was positive for some individuals who felt it allowed adaptations and their needs to be met. However, for others, disclosing their illness led to feelings of resentment, especially when employers had a poor attitude or lack of understanding towards the illness
	Wåhlin et al. (2019)	Sweden	To explore disease-related worries in persons with Crohn’s disease	12 participants aged 20–60 + (4 male, 8 female)	Interviews	Qualitative content analysis	Participants felt a need to talk about their IBD and the worry associated with it. They wanted to talk to someone in a similar situation to themselves
	Wang et al. (2023)	China	To explore the psychosocial process of posttraumatic growth in Chinese patients with CD	19 participants with CD (8 female, 11 male)	Interviews	Constructivist grounded theory	People’s perceptions of how the diagnosis of IBD made them look prevented people from disclosing their disease. This was particularly present in environments where "looking weak" put you at a disadvantage
	Woodward et al. (2016)	UK	The aim of this study was to detect IBD-specific distress and to generate items for a new IBD-distress scale	52 adult participants aged 17+	Secondary interview transcripts and an IBD focus group	Thematic analysis	Most participants agreed that discussing their diagnosis was a taboo subject and that the stigma associated with it made it difficult for them to talk about their experiences
	Zigron and Bronstein (2018)	Israel	To examine the activity of virtual health communities for users with IBD by understanding the role that these online spaces play as sources for information and social support	23 participants (15 female and 8 male) aged between 20 and 40	Interviews	Content analysis	The virtual community allows people to disclose personal information about their disease as it is viewed as “low risk” compared to disclosure in other situations. The use of disclosure in these communities was viewed positively by people wanting to seek or share information about IBD

### Quality Appraisal

Overall, study quality was considered moderate to high (see Supplementary Material), with all papers explicitly stating their aims and using appropriate qualitative methodology. However, reflexivity was identified as a weakness across most studies, while the description of data analysis was also not fully detailed for some papers.

### Thematic Synthesis

**Fig. 2:**
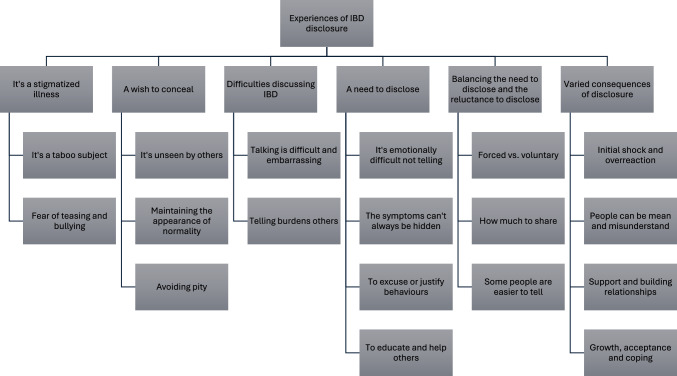
Thematic map

In total, the thematic synthesis generated six themes and 18 subthemes to describe the experiences of disclosing IBD (Fig. 2). These are detailed below, with references to subthemes indicated by italics. The studies and example quotes contributing to each theme/subtheme are presented in Table 3.

**Table 3:** Studies and quotes contributing the theme and subthemes

Theme	Subtheme	Example Quotes	Papers contributing to this theme
It’s a stigmatised illness	It’s a taboo subject	“*They can be such disgusting aspects, having diarrhoea and the other things, I just think, do people really want to hear about that, about someone else?"* *"Everyone gets a little squeamish when you start talking about your bowel habits."*	2; 5; 8; 9; 14; 15; 17; 22; 23; 27; 28; 33; 34
	Fear of teasing and bullying	*"I’m afraid to tell my friends about the disease. I’m afraid they will laugh."* *“I didn’t want them [peers] to hear […] because some stupid stuff might happen […] like making nasty remarks about it.”*	1; 14; 17; 26; 27
A wish to conceal	It’s unseen by others	*"…relative invisibility of IBD allowed participants to* *keep their condition successfully concealed much of the time."* *"it’s something for me that’s private."*	2; 6; 8; 12; 13; 14; 15; 27; 29; 33
	Maintaining the appearance of normality	*"They might not know the illness very well, and I didn’t want to make myself seem particularly different because of the illness."* *“I don’t want things to really change, where my friends think […] that all I like to talk about is my illness, because that’s all I can talk about."*	1; 2; 3; 5; 6; 8; 14; 19; 22; 23; 28; 32; 33
	Avoiding pity	*“Sometimes I don’t want people to know I’ve got an illness. I don’t want people to start the whole pity party, you know, ‘Oh, you poor thing! I feel really sorry for you.’"* *“Sometimes it feels like I’m being handled with kid gloves.”*	2; 3; 14; 23; 30
Difficulties discussing IBD	Talking is difficult and embarrassing	*"It was very difficult for me to explain, to cross this line and say that I’ve got this condition, and how I feel and everything. It took me a lot of time because it was not easy to explain how it is."* *“I find it’s, I don’t know, may be a bit embarrassing sometimes.”*	1; 2; 5; 7; 8; 10; 11; 12; 14; 15; 21; 22; 25; 27; 28; 33
	Telling burdens others	*"I don’t want to make a lot of noise… (because) I’m a burden on my family."* *" I always felt like it was going to fail, that it was going to be something. I say too much, and he would just leave."*	2; 3; 7; 14; 15; 17; 22; 30; 34
A need to disclose	It’s emotionally difficult not telling	*"I didn’t tell anyone. I hid that for years, believe it or not. And that was agony."* *"I think if I had tried to hide all the time, the stress levels would just make it so much worse."*	2; 7; 9; 14; 24; 25; 32; 33
	The symptoms can’t always be hidden	*"I talk about it less now, because in primary school, I couldn’t really hide it, because I couldn’t eat."* *"They would clearly know that something was wrong, and I couldn’t keep that from them anymore."*	1; 2; 7; 8; 13; 14
	To excuse or justify behaviours	*"I would have told him [referring to her husband] because he was thinking that I was making up excuses."* *"I fear the regular days off I need for treatment or sudden emergency flares may be misconstrued as skipping work without reason. And this could be another reason for my employer to fire me."*	1; 3; 8; 12; 26; 29; 30
	To educate and help others	*"I can help somebody else with questions. Somebody needing help in something I had trouble with and found a way to help to make it work. I love sharing any of that if someone asks."* *"Telling people] was definitely a challenge. I think it’s better when you explain it to people, because then they understand.”*	1; 2; 5; 8; 16; 18; 29; 30; 34
Balancing the need to disclose and the reluctance to disclose	Forced vs voluntary	*"I talk about it less now, because in primary school, I couldn’t really hide it, because I couldn’t eat."* *“Some people might come up to me after class and go, ‘Oh Will, why are you allowed to go to the toilet?’, and then I’d have to tell them. Well, I wouldn’t have to tell them, but I’d feel like, I was lying to them.”*	1; 2; 5; 8; 11; 12; 14; 29; 34
	How much to share	*"because it’s just telling someone about […] a part of you […], it’s easier telling people like what I have to have done.”* *"…just like because I wasn’t sure if if I’d like said something that wasn’t actually how like if I said it was bad when it wasn’t actually so bad or something."*	1; 2; 11; 14; 15; 27
	Some people are easier to tell	*"like an actual friend that I know that won’t tease me about it or something like that so umm yeah I have really good friends that they all know that I have Crohn’s."* *"So, uh, I couldn’t trust him; so I didn’t share anything with him.”*	1; 2; 4; 7; 11; 14; 17; 18; 21; 23; 24; 27; 31; 34
Varied consequences of disclosure	Initial shock and overreaction	*"When it’s explained to them, they either don’t take it seriously at all or they are profoundly shocked."* *"new people’s reactions that are the weirdest (.) it’s like oh my god (.) at work and stuff they just don’t get it the comment I had yesterday was ‘isn’t it really sad you’re so young’ and it’s like ‘so young what?’ and they’re like ‘so young to be like this’ and I don’t think like that."*	1; 2; 3; 7; 8; 11; 14; 17; 19; 21; 26; 27
	People can be mean and misunderstand	*“They [peers] would say things like, “You’re a bit like a cripple really, aren’t you?” and […] then, they would start talking about bowel movements. I could take all of the other things but, for some reason, them [sic] making comments associated with bowel movements, that really upsets me. That’s too much for me to deal with.”* *“some people think that because it’s a disease, Crohn’s—they say ‘Oh my God can I catch it off you?”*	1; 2; 3; 5; 6; 7; 11; 14; 17; 19; 21; 25; 26; 27; 29
	Support and building relationships	*“It [talking to friends] feels good because […] I know that they would listen to me and I know I can speak to someone about it and they won’t go telling other people that I don’t want to know.”* *"it helps a lot to talk about (IBD) with someone who has it"*	1; 2; 4; 5; 7; 8; 11; 13; 14; 16; 17; 18; 19; 20; 24; 29; 31; 32; 33; 34
	Growth, acceptance, and coping	*"You kind of have that freedom once you tell people. You don’t have to hide it anymore"* *"Because my parents and I talk things through about my IBD, I can deal with it"*	1; 2; 3; 7; 8; 14; 15; 17; 22; 28; 30; 34

#### Theme 1: It’s a Stigmatised Illness

The first analytic theme described how participants perceived their diagnosis within the context of society, the response they expected from others, and the impact this had on whether they disclosed their diagnosis. Participants described their IBD as *a taboo topic* and that talking about it was an “unacceptable conversation” due to the “privacy” of bowel habits both within society and within the home. Participants disclosure decisions were influenced by the perception that others are “squeamish” or “disgusted” (Dibley et al., 2020; Hall et al., 2005; Nicholas et al., 2007; Robertson et al., 2022; Saunders, 2014) by the topic of conversation, making individuals feel that IBD was “not allowed” to be talked about (Dibley et al., 2020; Gelech et al., 2021). Due to the perceived social stigma around bowel habits, participants described a *fear of teasing and bullying* if they disclosed IBD*,* as “bowel movements” are typically a source of humour in social situations and within the media.

#### Theme 2: A Wish to Conceal

The “invisibility” of IBD was identified as a barrier to disclosure due to it being *unseen by others* and therefore something that should remain concealed and kept hidden (Gelech et al., 2021). As their IBD was frequently already “invisible”, participants felt that it should remain a “private” illness which is “no one’s business” (Carter et al., 2020; Lolli, 2022; Micallef-Konewko, 2013; Savard & Woodgate, 2009; Woodward et al., 2016).

How individuals were viewed by others was a key barrier to disclosing IBD, with many participants avoiding disclosure to *maintain the appearance of normality*. It was a frequent experience that individuals living with IBD wanted to be seen as “normal” (Gelech et al., 2021; Micallef-Konewko, 2013; Rouncefield-Swales et al., 2020) and did not want to “become their disease” (Barned et al., 2016; Micallef-Konewko, 2013; Rouncefield-Swales et al., 2020). Participants also refrained from disclosure due wanting to *avoid pity*, including avoiding being made a fuss of or being viewed as weak.

#### Theme 3: Difficulties Discussing IBD

Many participants described avoiding IBD disclosures because *talking is difficult and embarrassing*, with many not knowing how to initiate conversations (Kluthe et al., 2018; Woodward et al., 2016), articulate information, or “find the right words” (Restall et al., 2016). Participants also avoided talking about their IBD as avoiding conversations was perceived as better than having to endure the uncomfortable emotions, and the emotional efforts, caused by disclosure.

As well their own emotions, individuals identified that in relation to their IBD disclosures, *telling burdens others*, leading to individuals frequently avoiding disclosure about their IBD diagnosis or experiences or “downplay[ing] its seriousness” (Carter et al., 2020) to protect others and prevent them from having to worry. However, in one study (Micallef-Konewko, 2013), participants recognised that disclosure may reduce others’ worry, as it enables people to know the truth and prevents them thinking it’s something more sinister.

#### Theme 4: A Need to Disclose

Although individuals may not want talk about their IBD, many participants identified that it was not always possible to avoid discussing it or keep their IBD hidden. Due to the symptoms associated with IBD, including diarrhoea and an urgency to use the toilet, participants identified that *it’s emotionally difficult not to tell* as trying to hide their illness caused stress, anxiety and social isolation (Gelech et al., 2021). Additionally, participants reported that their *symptoms can’t always be hidden,* as symptoms such as more frequent bathroom trips or weight/skin changes made the illness more “visible”. At these times, disclosure was deemed more of a necessity and enabled others to understand the reason for these observed changes (Frohlich, 2014). Additionally, participants felt the need to disclose *to excuse or justify behaviour,* avoid judgements from others and stop people “jumping to conclusions” due to individual’s “looking healthy” (Gelech et al., 2021; Matini & Ogden, 2016; Schwenk et al., 2014). Within the workplace, disclosure provided an “excuse” for “sick days” (Colmer, 2021; Vaughan & Jolliffe, 2023) or justified eating habits, as participants food choices received judgements for not being “healthy” or “appropriate” within a social context (Lolli, 2022).

Participants identified that disclosure became necessary to *educate and help others*. Due to the stigma associated with the disease, participants felt obliged to disclose to challenge the misconceptions held by the public (Dibley et al., 2017) and help increase others understanding of IBD (Carter et al., 2020; Schwenk et al., 2014), especially when the illness is confused with irritable bowel syndrome (Vaughan & Jolliffe, 2023). Due to the misunderstanding of IBD, participants spoke of learning about their disease following diagnosis, leading to them feeling obliged to pass on the information they had developed to help and support others who were trying to acquire an understanding for themselves.

#### Theme 5: Balancing the Need to Disclose and the Reluctance to Disclose

This theme encompassed the experiences and contextual factors influencing individuals’ decisions when faced with whether to talk about or conceal their IBD. Participants identified the difference between *forced versus voluntary disclosures,* with voluntary disclosures being more likely when individuals needed help/support, or when they felt in control of their symptoms. Participants reported that the “visibility” of their illness contributed to their disclosure decisions, with the presence of symptoms sometimes leading them to feel more forced into disclosure. For example, when other people directly asked questions or showed curiosity, participants felt that they would “*have to tell them”*. Others described feeling that they had more choice over disclosure, including choosing to delay disclosure as it would be easier to talk about IBD after the “bad symptoms” had passed (Carter et al., 2020). However, even when participants made voluntary disclosures, follow-up questions or people bringing up IBD could make disclosure feel more forced (Colmer, 2021; Lolli, 2022).

Regardless of whether disclosure felt forced or voluntary, participants had to decide *how much to share,* including whether to make a “complete disclosure” or “selective disclosure” (Carter et al., 2020; Ruan & Zhou, 2019). Deciding the amount of information to share influenced people’s experience of how successful their disclosure was, with some feeling regret around withholding information (Micallef-Konewko, 2013). Disclosure decisions were also influenced by the audience, with *some people being easier to tell.* Participants identified the importance of “trusting” others to tolerate and respond positively to the information that is shared (Barned et al., 2016; Restall et al., 2016; Rouncefield-Swales et al., 2020), with disclosure to others living with IBD feeling easier due to the shared understanding of the challenges (O’Leary et al., 2020; Zigron & Bronstein, 2018). However, the decision regarding who to tell varied across the lifespan, with younger participants being more selective, due to fears that their personal information would be spread.

#### Theme 6: The Varied Consequences of Disclosure

In contrast to earlier themes which identified individual’s perceptions about potential consequences of disclosure that acted as barriers to disclosure, Theme 5 describes the direct experiences participants had when making disclosures. Within this theme, participants described that disclosure was frequently met with *shock or overreaction,* with others showing pity, extreme worry, and negativity (Micallef-Konewko, 2013; Palant & Himmel, 2019; Saunders, 2014). Disclosure was also experienced negatively because it was frequently met by others being *mean or misunderstand* the illness, including others making comments that the individual “looks fine”, comparing their experiences to others with IBD, or making comments about them being “contagious”. Although these comments were considered negative, there were no direct reports of individuals experiencing bullying because of their IBD.

While such negative reactions were often experienced in the short term, in general, participants described positive disclosure experiences which enabled them to receive *support and build relationships*, as others generally wanted to help and understand. Participants also experienced disclosing their IBD as important on a personal level, with it contributing to them feeling relieved, free, happy, and less embarrassed by their illness, which allowed them to *grow, accept and cope* with their IBD, including enabling participants to feel in control and able to cope with their IBD across social contexts.

## Discussion

To the authors’ knowledge, this is the first review to systematically synthesise experiences of self-disclosure within an IBD population across the lifespan. A total of 34 studies contributed to five themes which identified some of the processes and experiences involved in individuals’ disclosure decisions.

Consistent with the Disclosure Processes Model (DPM; Chaudoir & Fisher, 2010), this review highlights the complex processes individuals with IBD encounter in relation to self-disclosure. Participants identified an integration of factors, both on a personal and societal level, that contribute to their feelings towards disclosure and whether they take this step. The meta-synthesis themes suggest there were possibly different motivators for disclosure amongst participants, which corresponded with the “approach-goals” and “avoidance-goals” theorised by the DPM (Chaudoir & Fisher, 2010). For example, in the theme ‘*a need to disclose*’, participants described experiencing disclosure as an opportunity to educate others (Carter et al., 2020; Schwenk et al., 2014; Vaughan & Jolliffe, 2023) and provide support to others (Carter et al., 2020; Nehasil, 2014). These “approach-goals” appear to facilitate individuals’ disclosure due to the perceived benefits that it would have on other people, resulting in generally positive attitudes towards disclosure.

Additionally, “avoidance-goals” appeared to facilitate individual’s disclosure. For example, in the theme ‘*a need to disclose*’, some participants described that their disclosure was motivated by trying to justify their behaviour and avoid negative judgements from others (Colmer, 2021; Lolli, 2022; Vaughan & Jolliffe, 2023). Avoidance-goals could also act as a barrier to disclosure, with individuals choosing not to disclose their IBD in attempts to avoid perceived negative responses from others, such as *teasing and bullying* and *pity* (Barned et al., 2016; Carter et al., 2020; Micallef-Konewko, 2013; Sammut et al., 2017)*.* This appears consistent with findings within the DPM literature on other physical health conditions, including HIV (Chaudoir, 2009; Chaudoir & Fisher, 2010; Chaudoir et al., 2011; Krsmanovic & Dean, 2022).

In line with findings from other chronic illnesses (Benson et al., 2015; Chaudoir et al., 2011; Frank et al., 2006; Joachim & Acorn, 2000) and previous reviews in IBD (Guo et al., 2020; Micallef-Konewko, 2013; Muse et al., 2021; Taft & Keefer, 2016), the current review appears to support the role of stigma, or perceived stigmatisation, as a barrier to disclosure, with the themes *it’s a stigmatised illness* and *a wish to conceal* describing how participants perceived IBD to be viewed as “taboo” and “inappropriate” to talk about within the wider society.

Aligning with Micallef-Konewko (2013), this review highlights that disclosure decisions are influenced by more than just stigma, with visibility, personal emotions, and wanting to maintain a “normal” life impacting on whether disclosure took place. In addition, the current review considers both paediatric and adult literature, offering a broader understanding of disclosure experiences in this population which supported factors previously identified in other chronic illnesses, including diabetes (Ledford et al., 2022). Although similar challenges of self-disclosure arose, there appeared key differences across the lifespan, with children experiencing more fear of bullying and teasing, while adults identified the benefits of obtaining support, especially in the workplace.

Alongside disclosure decision-making processes, this review highlights the experiences encountered when IBD disclosures are made. For some individuals, disclosure was identified as a potentially harmful action, which could lead to harmful comments or continued misunderstandings (Dibley et al., 2020; Micallef-Konewko, 2013; Schwenk et al., 2014). However, multiple studies reported positive outcomes, with disclosure being important for connecting with others, building stronger relationships, and accessing help and support (Carter et al., 2020; Frohlich, 2014; Micallef-Konewko, 2013). Disclosure was also identified as an important step in individuals’ IBD journey, with disclosure facilitating general acceptance of oneself and IBD.

### Implications of Findings

This review also suggests that, despite the frequently positive impact of IBD disclosure on coping and interpersonal relationships, disclosure remains avoided by some due to IBD being a “not talked about” and misunderstood topic within wider society. This suggests that healthcare professionals and charitable organisations should continue with, and expand, their efforts to increase society’s understanding of IBD (IBD UK, n.d.). By encouraging conversations around IBD at a more systemic level, it may become a more open, acceptable, and less stigmatised topic of conversation, making disclosure easier (Taft et al., 2017). Additionally, there appears to be a role for clinical health psychologists and community psychology in supporting individuals living with IBD in relation to disclosure and in destigmatising IBD as a health condition within society (Phelan et al., 2023).

As IBD can have an early onset, with between 10 and 20% of individuals receiving a diagnosis before the age of 18 (Wilson & Russell, 2017), it would seem best that efforts to increase societal awareness also include children. Schools may benefit from receiving workshops and teacher training, facilitated by healthcare professionals or charities that raise awareness of IBD (Kim et al., 2019).

The barriers to disclosure, such as perceived stigma, may also influence the information some patients share with their clinicians, which may contribute to their needs not being fully met (Nuttall, 2019; Schreiber et al., 2012). Therefore, clinicians should consider how they are supporting their patients to discuss their difficulties and ensure that they offer the opportunity for disclosure in a safe, non-judgemental way. Furthermore, healthcare professionals may be able to support individuals to consider their disclosure decisions more generally. Supporting individuals with this may contribute to addressing the stress associated with non-disclosure, which may allow for a better quality of life and disease outcomes (Boye et al., 2011; Sainsbury & Heatley, 2005).

### Limitations

Although this is the first review to meta-synthesise experiences, barriers and facilitators relating to IBD disclosure that go beyond stigma and also the first to synthesise this across the life span, there are important limitations that must be acknowledged. Although a relatively large number of studies (*n* = 34) have been included in this review, the majority of these had a broader focus than disclosure and hence only contributed limited data, while the six studies that most closely focussed on disclosure contributed the most to the meta-synthesis. Therefore, despite a relatively high number of included articles, the amount of data extracted and analysed from some studies was limited, with most data being extracted from the papers which explored disclosure directly.

Second, although the overall quality of studies was generally rated as moderate to high, reflexivity was a weakness across most, with many researchers not detailing their position/experiences and how this influenced their approach to research or the interpretation of the research.

Third, it is important to acknowledge that most of the included studies did not report the ethnicity of their participants and, in those that did, the majority were Caucasian. Therefore, the finding may not be transferrable to all populations living with IBD, especially as perceptions of “normality” and perceived/internalised stigma may vary cross-culturally (Burns, 2003; Franz et al., 2013; Wong et al., 2017).

Finally, it is acknowledged that individuals’ experiences of IBD and self-disclosures may depend on the extent of their digestive symptoms, which are often higher in individuals with Crohn’s disease compared to Ulcerative Colitis (Bergeron et al., 2018). However, the included studies did not always indicate the diagnosis within their qualitative, making it difficult to understand the impact of IBD diagnosis on self-disclosure. Future research would benefit from further examining whether the experiences, barriers or facilitators of disclosure varied between IBD diagnosis, to ensure support is meeting individuals’ specific needs.

## Conclusions

This meta-synthesis of people’s experiences of disclosing IBD appears to illustrate how individuals balance a reluctance to disclose, connected to stigma and negative responses from others, with the positive benefits of disclosure and/or a need to disclose. It highlights the potential complexity of disclosure decisions and the possible interaction of personal and societal factors on them. Further research is needed to develop an understanding of IBD disclosure across different cultures and different contexts, including school, work, relationships, friendships, and medical settings.

## Supplementary Information

Below is the link to the electronic supplementary material.

Supplementary file1 (DOCX 27 KB)

## Data Availability

No datasets were generated or analysed during the current study.
