# Supplementary material for: Disclosing Inflammatory Bowel Disease: A Systematic Review and Meta-Synthesis Exploring the Experience of, and Barriers and Facilitators to, Self-Disclosure
Source: J Clin Psychol Med Settings. 2025 Jun 2;32(3):526–48. doi: 10.1007/s10880-025-10079-z (PMC12370872; doi:10.1007/s10880-025-10079-z)
Supplement: Supplementary file 1 — Supplementary file1 (DOCX 27 KB) [file 10880_2025_10079_MOESM1_ESM.docx]

**Supplementary Material**

1. **Pre-planned search terms**

| **Search Area** |  | **Terms used** |
| --- | --- | --- |
| Inflammatory Bowel Disease |  | (“Inflammatory Bowel Disease*” OR IBD OR Crohn* OR Colitis) |
|  | *AND* |  |
| Disclosure |  | (disclos* OR self-disclos* OR shar* OR tell* OR conceal* OR talk* OR experience* OR expos*) |
|  |  |  |
| Qualitative literature | *AND* | (qualitative OR interview* OR IPA OR “interpretive phenomenological analysis” OR narrative* OR “focus group*” OR “thematic analysis” OR “grounded theory” OR “discourse” OR “ethnography”) |

1. **Complete Quality Appraisal**

The quality of included studies was assessed by the author and an independent reviewer using the CASP qualitative assessment. A summary of the quality appraisals is shown in Table 4 and in full in Appendix E.

## ***Aims and Design***

All studies provided a clear statement of their aims, objectives, or research question/s. Whilst the aims and objectives of each study varied, all aimed to understand or describe complex phenomena, predominantly the experiences of IBD, thus qualitative methods were deemed appropriate. There were several studies (n=9) who did not provide details regarding the research design. However, the design was identifiable from other sections of the paper despite it not being explicit.

## ***Participants and Sampling***

Most studies provided clear and appropriate information on how participants were recruited, including the methods or organisations used to support this, an in-depth inclusion/exclusion criterion for participants and information about the demographics of their sample. However, it was acknowledged that very few studies provided details on why their sample was chosen and what knowledge this offered. Although not directly measured by the CASP, it was identified that ethnicity was not reported in 24 papers, raising caution regarding the studies’ transferability.

## ***Ethical Considerations and reflexivity***

Ethical considerations were a strength across the studies as all stated having obtained ethical approval from appropriate panels/organisations. However, it is acknowledged that the level of detail provided around ethical considerations varied across studies. Most studies provided details on obtaining informed consent from participants (n=26) and several provided details around confidentiality (n=9).

Although the consideration of researcher positioning and bias varied across studies, it was generally assessed as the weakest domain with only nine papers providing detailed consideration of their role and position, including personal diagnoses of IBD or gender, and its influence on the qualitative approach used. Therefore, caution should be taken in interpreting the studies, as it is uncertain how the researchers positioning may have influenced their data collection and/or findings.

## ***Data Collection and analysis***

Details regarding data collection varied but was assessed to be adequate across all studies. Most studies used a form of interview, however, only 5 papers provided full interview schedules.

Information related to data analysis varied, but methods were assessed as appropriate across studies. A high proportion of studies reported the process of coding, theme development and/or consideration of rigor in data analysis. However, a few studies reported themes without detailing the development of these. Some papers (n=15) referred to saturation, with two justifying saturation to be impossible within their chosen theoretical framework (Dibley et al., 2017; Dibley et al., 2020).

## ***Findings and Value***

Although the findings varied in the level of detail provided, all studies presented clear information about their themes with supporting quotes. However, it was recognised that the discussion around conflicting findings was limited across all studies. Therefore, there should be caution in interpreting these results, as it is unclear what additional findings may have emerged and the impact this may have had on the findings. There was variation in the amount of information provided regarding the value of the research, however most studies met at least one criterion. Most studies discussed the implications of their research, including how findings could be applied to healthcare and made recommendations for future research.

***Summary of quality appraisal***

Overall, study quality was considered moderate to high, with all papers explicitly stating their aims and using appropriate qualitative methodology. However, it was recognised that reflexivity was a weakness across most studies. It was also identified that descriptions of data analysis were not fully detailed for some papers. The quality of these papers was considered throughout the thematic analysis and as seen in Table 5.

1. **Quality rating per study per criterion**

| **Author** | **Aims** | **Methods** | **Research Design** | **Recruitment** | **Data collection** | **Reflexivity** | **Ethical Issues** | **Data analysis** | **Findings** | **Research Value** |
| --- | --- | --- | --- | --- | --- | --- | --- | --- | --- | --- |
| Barned et al. (2016) | Y | Y | Y | Y | Y | P | Y | P | Y | Y |
| Murphy et al. (2022) | Y | Y | Y | P | Y | Y | Y | Y | Y | Y |
| Savard & Woodgate (2009) | Y | Y | Y | Y | Y | N | Y | Y | Y | Y |
| Saunders (2014) | Y | Y | Y | Y | Y | P | Y | P | Y | P |
| Frohlich (2012) | Y | Y | P | Y | Y | Y | Y | P | Y | P |
| Devlen et al. (2014) | Y | Y | P | Y | Y | N | Y | Y | Y | Y |
| Sammut et al. (2017) | Y | Y | Y | Y | Y | P | Y | Y | Y | Y |
| Robertson et al. (2022) | Y | Y | Y | Y | Y | Y | Y | Y | Y | Y |
| Dibley et al. (2017) | Y | Y | Y | Y | Y | P | Y | Y | Y | P |
| Hall et al. (2005) | Y | Y | P | Y | Y | N | Y | Y | Y | Y |
| O'Leary et al. (2020) | Y | Y | Y | Y | Y | N | Y | Y | Y | Y |
| Gelech et al. (2021) | Y | Y | Y | Y | Y | P | Y | Y | Y | Y |
| Restall et al. (2016) | Y | Y | Y | Y | Y | P | Y | Y | Y | Y |
| Dibley et al. (2020) | Y | Y | Y | Y | Y | P | Y | Y | Y | Y |
| Zigron & Bronstein (2018) | Y | Y | P | Y | Y | Y | N | Y | Y | Y |
| Matini & Ogden (2016) | Y | Y | N | Y | Y | N | Y | Y | Y | P |
| Palant & Himmel (2018) | Y | Y | Y | P | Y | N | Y | Y | Y | P |
| Salazar & Heyman (2014) | Y | Y | Y | P | Y | N | Y | Y | Y | P |
| Nicholas et al. (2007) | Y | Y | Y | P | Y | N | Y | Y | Y | P |
| Schwenk et al. (2014) | Y | Y | Y | Y | Y | Y | Y | P | Y | Y |
| Nehasil (2014) | Y | Y | Y | Y | Y | Y | Y | Y | Y | Y |
| Woodward et al. (2016) | Y | Y | Y | P | Y | P | Y | Y | Y | Y |
| Kluthe et al. (2018) | Y | Y | Y | Y | Y | N | Y | Y | Y | Y |
| Ruan & Zhou (2019) | Y | Y | Y | Y | Y | Y | Y | P | Y | Y |
| Vaughan & Jolliffe (2023) | Y | Y | Y | Y | Y | Y | Y | Y | Y | Y |
| Micallef-Konewko (2013) | Y | Y | Y | Y | Y | Y | Y | Y | Y | Y |
| Carter et al. (2020) | Y | Y | Y | Y | Y | N | Y | P | Y | P |
| Lolli (2022) | Y | Y | Y | P | Y | Y | Y | Y | Y | Y |
| Kitchen et al. (2020) | Y | Y | P | Y | Y | N | Y | Y | Y | Y |
| Wang et al. (2023) | Y | Y | Y | Y | Y | Y | Y | Y | Y | Y |
| Rouncefield-Swales et al. (2020) | Y | Y | P | Y | Y | N | Y | Y | Y | Y |
| Peters & Brown (2022) | Y | Y | Y | Y | Y | N | Y | Y | Y | Y |
| Colmer (2021) | Y | Y | P | P | Y | N | Y | Y | Y | Y |
| Wåhlin et al. (2017) | Y | Y | Y | P | Y | N | Y | Y | Y | Y |

Y = Criterion met, P = Criterion partially met, N = Criterion not met

1. Researchers’ reflexive account

Throughout the thematic analysis, the authors took a reflexive position which enabled them to think about assumptions and expectations about the process, data, and themes. This helped them to challenge previous assumptions around IBD and thematic analysis, enabling them to consider the data more holistically and generate themes that emerged within it. The reflexive position helped the authors to remain aware of their prior knowledge, assumptions and experiences and enabled them to consider the influence of these on processes throughout the analysis.

Prior to starting this review, the authors had mixed experiences of professional experiences working with IBD and personal experience of living with, or supporting others, with invisible Chronic illnesses. It is acknowledged that these would have shaped the expectations the authors held. However, due to varying experiences, it was felt that the discussions between authors and reflection throughout the process limited the impact these prior experiences had on the analysis and results.

Due to prior experiences and knowledge of the area, the authors expected that the role of stigma would be a key factor in disclosure, especially due to the taboo nature of the IBD symptoms. From the authors experiences, it was also expected that disclosure decisions would be motivated by avoiding judgements for things such as work absences or judgements from others. Based on the authors experiences, it was expected that experiences of disclosure would be motivated by individuals’ perceptions of how other see them and influenced by more societal factors.
